# Cezanne regulates E2F1-dependent HIF2α expression

**DOI:** 10.1242/jcs.168864

**Published:** 2015-08-15

**Authors:** Sonia Moniz, Daniel Bandarra, John Biddlestone, Kirsteen J. Campbell, David Komander, Anja Bremm, Sonia Rocha

**Affiliations:** 1 Centre for Gene Regulation and Expression, College of Life Sciences, University of Dundee, Dundee DD1 5EH, UK; 2 Beatson Institute for Cancer Research, Glasgow G61 1BD, UK; 3 Medical Research Council Laboratory of Molecular Biology, Francis Crick Avenue, Cambridge Biomedical Campus, Cambridge CB2 0QH, UK; 4 Buchmann Institute for Molecular Life Sciences, Goethe University Frankfurt, Max-von-Laue-Strasse 15, Frankfurt am Main 60438, Germany

**Keywords:** HIF2α, Cezanne, Hypoxia, Cell cycle, E2F1, ChIP

## Abstract

Mechanisms regulating protein degradation ensure the correct and timely expression of transcription factors such as hypoxia inducible factor (HIF). Under normal O_2_ tension, HIFα subunits are targeted for proteasomal degradation, mainly through vHL-dependent ubiquitylation. Deubiquitylases are responsible for reversing this process. Although the mechanism and regulation of HIFα by ubiquitin-dependent proteasomal degradation has been the object of many studies, little is known about the role of deubiquitylases. Here, we show that expression of HIF2α (encoded by *EPAS1*) is regulated by the deubiquitylase Cezanne (also known as OTUD7B) in an E2F1-dependent manner. Knockdown of Cezanne downregulates HIF2α mRNA, protein and activity independently of hypoxia and proteasomal degradation. Mechanistically, expression of the HIF2α gene is controlled directly by E2F1, and Cezanne regulates the stability of E2F1. Exogenous E2F1 can rescue HIF2α transcript and protein expression when Cezanne is depleted. Taken together, these data reveal a novel mechanism for the regulation of the expression of HIF2α, demonstrating that the HIF2α promoter is regulated by E2F1 directly and that Cezanne regulates HIF2α expression through control of E2F1 levels. Our results thus suggest that HIF2α is controlled transcriptionally in a cell-cycle-dependent manner and in response to oncogenic signalling.

## Abstract (precis)

**Summary:** Cezanne regulates HIF2α levels and activity by modulating expression of the HIF2α gene through regulation of E2F1 protein levels. Cezanne depletion impairs normal cell cycle progression and increases cell death.

## INTRODUCTION

Adaptation to changes in the microenvironment requires tight regulation of gene expression. In response to low O_2_ levels, gene expression is mainly regulated by the hypoxia inducible factor (HIF) family of transcription factors, which enact a transcriptional programme that allows cell survival as well as the re-establishment of O_2_ supply. The de-regulation of HIF signalling has severe implications on several disease processes, including stroke, tissue regeneration and cancer (Semenza, 2012).

HIF is a heterodimeric transcription factor comprising one of three O_2_-labile α subunits (HIF1α, HIF2α or HIF3α; encoded by *HIF1A*, *EPAS1* and *HIF3A*, respectively) and an O_2_-insensitive β subunit (encoded by *ARNT*). So far, most studies regarding HIF regulation have been directed towards HIF1α, and little is known about how the other subunits are modulated. Interestingly, although HIF1α and HIF2α share sequence similarity and a number of transcriptional targets, the tissue distribution and functional properties of the two proteins are considerably different (Chiavarina et al., 2012; Gordan et al., 2007a). Furthermore, HIF2α has been associated with tumour-promoting properties in different types of tumours (Bangoura et al., 2007; Chiavarina et al., 2012; Holmquist-Mengelbier et al., 2006; Noguera et al., 2009; Raval et al., 2005; Scrideli et al., 2007).

HIF regulation by O_2_ impacts on the protein stability of the α subunits and is dependent on the activities of a class of dioxygenase enzymes called prolyl hydroxylases (PHDs). Under normal O_2_ tension, PHDs catalyse the hydroxylation of two prolyl residues on HIFα subunits in the O_2_-dependent degradation (ODD) domain (Fandrey et al., 2006). Prolyl-hydroxylation then attracts the von Hippel-Lindau (vHL) tumour suppressor protein, which recruits the elongin-C–elongin-B–cullin-2–E3-ubiquitin-ligase complex, leading to K48-linked polyubiquitylation and proteasomal degradation of HIFα (Ivan et al., 2001; Jaakkola et al., 2001; Yu et al., 2001). Under hypoxic conditions, PHDs, which require molecular O_2_ as a co-factor, become inactive allowing HIFα protein stability, dimerization with HIF1β and activation of transcriptional targets. More recently, additional degradation mechanisms have been described for HIFα subunits (Bento et al., 2010; Bremm et al., 2014; Hubbi et al., 2013; Li et al., 2007; Liu et al., 2015), emphasising the complexity of HIF homeostasis.

Deubiquitylases (DUBs) are enzymes that can contribute to the stabilisation of target proteins through the hydrolysis of ubiquitin chains, and many DUBs associate with E3 ligase complexes to fine-tune the ubiquitylation status of a common substrate (Komander et al., 2009). Although DUBs are starting to emerge as attractive therapeutic targets for diseases associated with deregulated protein expression levels, such as cancer (Fraile et al., 2012; Pal et al., 2014), little is known so far about the role of DUBs in the regulation of the HIF system. The ubiquitin specific proteases (USPs) USP20 and USP8 have been shown to deubiquitylate HIF1α (Li et al., 2005, 2002; Troilo et al., 2014), whereas USP19 stabilises HIF1α in a non-catalytic manner (Altun et al., 2012; Bett et al., 2013) and USP52 is required for stability of mRNA encoding HIF1α (Bett et al., 2013). Additionally, we have recently demonstrated that the ovarian tumour protease (OTU) DUB Cezanne (OTUD7B) binds to and regulates the turnover of HIF1α, thereby modulating HIF1 transcriptional activity (Bremm et al., 2014). However, there is no information on whether or how DUBs alter HIF2α protein.

Here we report that Cezanne regulates the expression and activity of HIF2α by modulating expression of the HIF2α gene. We show that Cezanne regulates E2F1 protein levels and that E2F1 is required for expression of the HIF2α gene – by directly binding to the HIF2α promoter. Furthermore, we demonstrate that the regulation of HIF2α through Cezanne is E2F1-dependent and that Cezanne depletion impairs normal cell cycle progression and increases cell death.

## RESULTS

The majority of research concerning the regulation of HIFs has been directed towards HIF1α. HIF1α is decorated by many post-translation modifications such as hydroxylation, sumoylation, ubiquitylation, nitrosylation and phosphorylation; however, whether all of these are also present on HIF2α is currently unknown. In addition, mechanisms controlling transcription and translation of the HIF1α gene have also been described (Linehan et al., 2010; Rius et al., 2008; van Uden et al., 2008). By contrast, very little information exists on how expression of HIF2α is controlled, apart from that on the canonical PHD–vHL degradation axis. Recently, we have found that HIF1α is regulated at the protein level by the deubiquitylase enzyme OTUD7B/Cezanne (Bremm et al., 2014). Because, currently, no DUB that regulates HIF2α has been identified, and considering the growing importance of DUBs in cancer, we decided to investigate if Cezanne alters the functions and levels of HIF2α.

### siRNA-mediated silencing of Cezanne results in decreased expression of the HIF2α protein

**Fig. 1.**
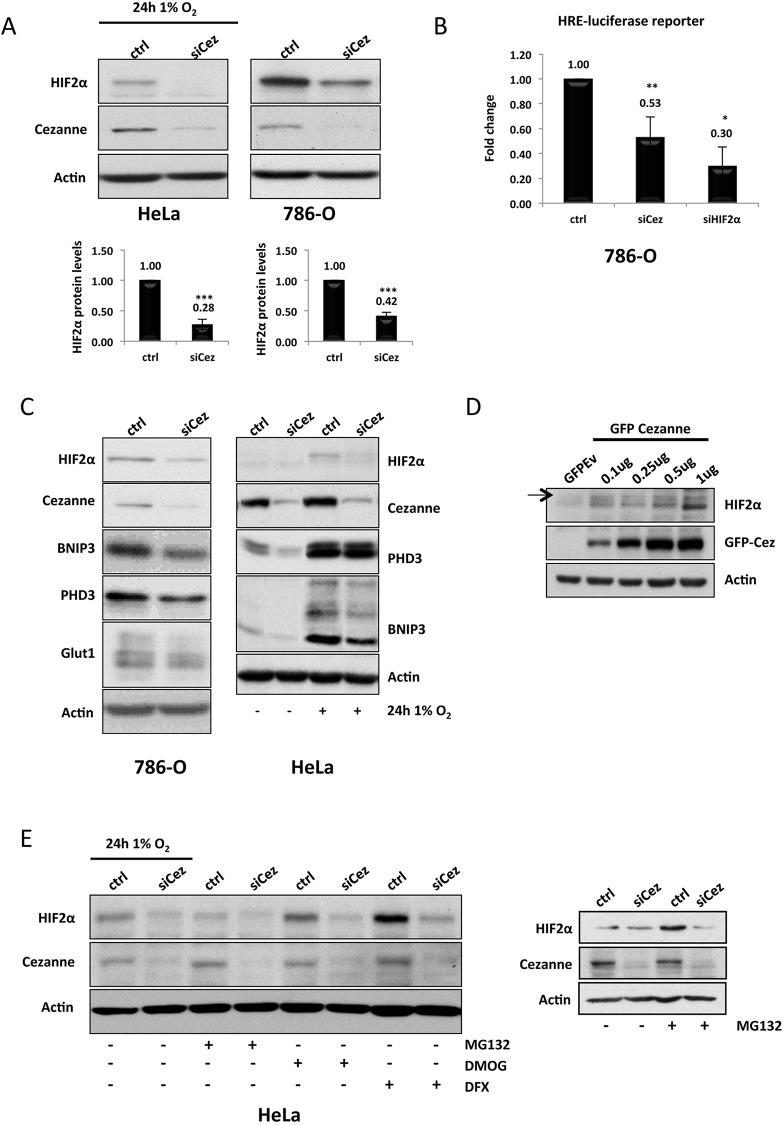
**Cezanne regulates HIF2α protein levels and activity in a PHD- and vHL-independent manner.** (A) HeLa and 786-O cells were transfected with either a non-targeting control (ctrl) or a Cezanne-targeting siRNA (siCez). At 24 h after transfection, HeLa cells were exposed to 1% O_2_, and whole cell lysates of both HeLa and 786-O cells were prepared after further 24 h incubation. Protein levels of HIF2α and Cezanne were assessed by western blotting. The band intensities were measured, and the values normalised to those of the control siRNA (*P*-values are significant according to the Student's *t*-test; ***P<0.001). (B) 786-O cells were co-transfected with a HRE-luciferase reporter plasmid and each of the siRNAs for 48 h. Results are mean±s.d. for at least three independent experiments expressed as fold activation or repression normalised to those of the control siRNA (*P*-values are significant according to the Student's *t*-test; *P<0.05, **P<0.01). (C) 786-O cells under basal conditions and HeLa cells, either under basal conditions or after exposure for 24 h to 1% O_2_, were transfected with control or Cezanne siRNAs, and whole cell lysates were prepared 48-h post-transfection and analysed by western blotting with the antibodies depicted. (D) HeLa cells were transfected with increasing amounts of a wild-type GFP–Cezanne plasmid, keeping the total amount of DNA in each transfection constant, and harvested for western blot analysis after 48 h. The arrow indicates HIF2α protein. (E) HIF proteasomal degradation pathways were inhibited in HeLa cells transfected with control or Cezanne siRNAs and exposed to 1% O_2_ for 24 h to stabilise HIF2α levels. Cells were treated with 20 µM MG132 for 3 h (left panel) or 10 µM MG132 for 7 h (right panel), 1 mM DMOG for 90 min or 200 µM DFX for 24 h.

To determine whether Cezanne alters expression of HIF2α in cells, we employed a loss-of-function approach using small interfering (si)RNA-mediated knockdown. HeLa cells were transfected with an siRNA specific for Cezanne for 48 h and incubated for 24 h under 1% O_2_ to allow HIF2α protein stabilisation before harvesting. Under these conditions, we could observe a clear and consistent reduction in HIF2α protein levels. The same effect could also be observed in the cell line 786-O, a renal cancer cell line that only expresses HIF2α and not HIF1α (Shen et al., 2011) (Fig. 1A). In 786-O cells, the analysis was performed under normal O_2_ conditions because these cells are vHL-negative and consequently express high levels of HIF2α under normoxia (Shen et al., 2011). We further tested whether the observed decrease in HIF2α levels correlated with decreased HIF activity. By co-transfecting 786-O cells with a hypoxia responsive element (HRE)-luciferase reporter, we observed that there was a significant reduction in HIF activity in cells that had been depleted of Cezanne, as shown in Fig. 1B. Additionally, both 786-O and HeLa cells showed reduced levels of known HIF targets, such as Glut1, PHD3 and BNIP3 (Fig. 1C). These results were also obtained by using a different siRNA against Cezanne (supplementary material Fig. S1A), indicating that the reduction of HIF2α levels following Cezanne depletion is not a result of siRNA off-target effects. Furthermore, when we performed gain-of-function experiments, where increasing concentrations of Cezanne were transfected into cells, HIF2α levels increased concomitantly (Fig. 1D).

Cezanne has been associated with hydrolysis of K11-linked ubiquitin chains (Bremm and Komander, 2011; Bremm et al., 2014), and HIF2α is known to be regulated by the proteasome (supplementary material Fig. S1B), as such, we next tested if depletion of Cezanne induces excessive proteasomal degradation of HIF2α. To this end, cells that had been depleted of Cezanne were treated with MG132, as well as DFX or DMOG (two PHD inhibitors), under hypoxia, and the levels of HIF2α were analysed. Interestingly, none of these treatments rescued HIF2α levels when Cezanne was depleted (Fig. 1E). Taken together, these data suggest that HIF2α is regulated by Cezanne in a PHD- and proteasome-independent manner. Interestingly, vHL inactivation was also unable to rescue the Cezanne-mediated reduction of HIF2α that was observed in 786-O and RCC4 cells (data not shown). This suggests that the mechanism by which Cezanne alters HIF2α levels is distinct from the mechanism by which Cezanne targets HIF1α, where we have previously observed a clear dependence on vHL (Bremm et al., 2014).

### siRNA-mediated silencing of Cezanne regulates the level of HIF2α transcripts

**Fig. 2.**
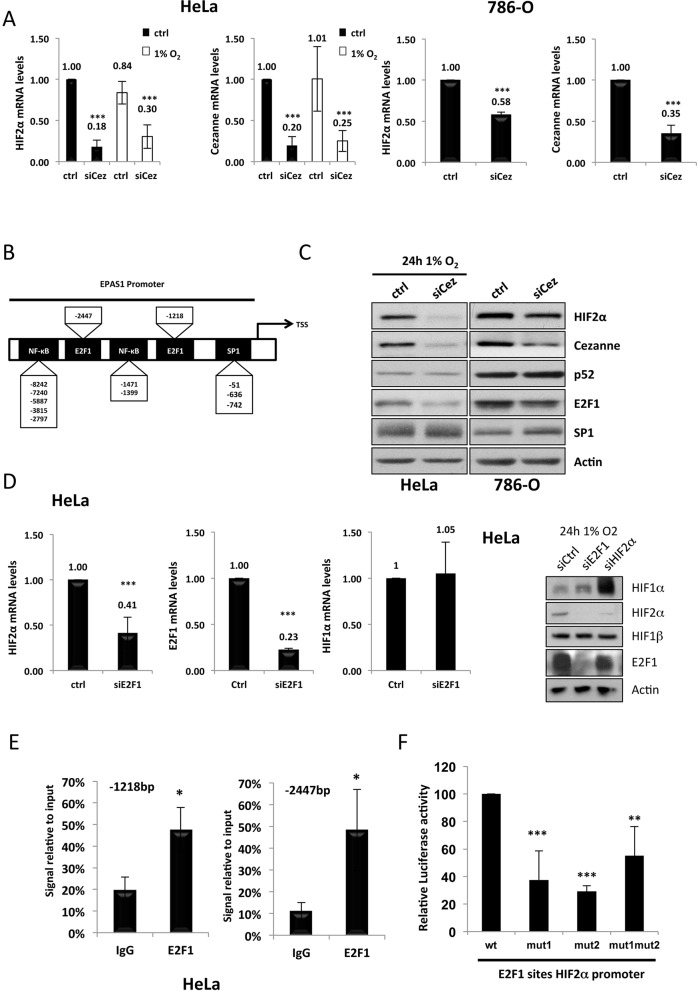
**Cezanne and E2F1 modulate HIF2α expression.** (A) HeLa and 786-O cells were transfected with control (ctrl) or Cezanne-targeting siRNAs (siCez), and whole cell lysates were prepared 48 h post-transfection, and total RNA was extracted. RT-qPCR was performed in order to analyse the mRNA levels of HIF2α and Cezanne using actin as a normalising gene (*P*-values are significant according to the Student's *t*-test; ***P<0.001). The fold-changes relative to the control are shown above the bars. (B) Schematic diagram depicting the results of the bioinformatic analysis of potential transcription factor binding sites (indicated) in the HIF2α gene promoter region. Base pairs locations are shown relative to the transcription start site (TSS). (C) 786-O cells under basal conditions and HeLa cells exposed for 24 h to 1% O_2_ were transfected with control or Cezanne-targeting siRNAs, and whole cell lysates were prepared 48 h post-transfection and analysed by western blotting with the antibodies indicated. (D) HeLa cells were transfected with control or E2F1-targeting siRNAs, and 48 h post-transfection, total RNA and protein extracts were prepared. RT-qPCR analysis of HIF2α, HIF1α and E2F1 mRNA was performed using actin as a normalising gene (*P*-values are significant according to the Student's *t*-test; ***P<0.001). Protein levels of HIF2α, HIF1α, HIF1β and E2F1 under the same conditions were also analysed by western blotting. The blot on the right is representative of this analysis. (E) ChIP analyses were performed in untreated HeLa cells using an antibody against E2F1 and a control IgG antibody. HIF2α promoter regions were amplified using specific primers, and the levels of E2F1 recruitment were analysed by using qPCR (*P*-values are significant according to the Student's *t*-test; *P<0.05). (F) HEK293 cells were transfected with 1 µg of each of the indicated luciferase constructs for 48 h before lysis and luciferase activity analysis (*P*-values are significant according to the Student's *t*-test; *P<0.05, **P<0.01, ***P<0.001). wt, wild-type.

HIF1α can be regulated at the transcriptional and translational level independently of PHD and vHL activity (Linehan et al., 2010; Rius et al., 2008; van Uden et al., 2008). Given our results above, we next investigated whether Cezanne depletion has an effect on the expression of the HIF2α gene. We could observe a marked and significant reduction in HIF2α mRNA in cells that had been depleted of Cezanne, independently of hypoxia, in both HeLa and 786-O cells (Fig. 2A).

Given that little is known about the regulation of the HIF2α gene, we performed a bioinformatic analysis of the HIF2α promoter in order to identify potential binding sites for transcription factors (Fig. 2B). This analysis identified several binding sites for transcription factors such as NF-κB, E2F1 and SP1 (Fig. 2B). To determine whether Cezanne downregulation affected the expression of any of these transcription factors, western blot analysis was performed. We could not detect any significant changes in the levels of SP1 or NF-κB subunits, such as p52 (encoded by *NFKB2*, which is then cleaved into p52), but depletion of Cezanne resulted in a reduction in E2F1 protein levels (Fig. 2C). Similar reduction in E2F1 protein levels was also observed when a second siRNA oligonucleotide was used to knockdown Cezanne (supplementary material Fig. S1A)

### E2F1 regulates HIF2α transcription

Our results indicated that Cezanne depletion results in a reduction of E2F1, a transcription factor that is predicted bioinformatically to control expression of the HIF2α gene. To investigate whether E2F1 can indeed control HIF2α, we next depleted E2F1 and analysed HIF2α mRNA levels (Fig. 2D). It was possible to observe that the knockdown of E2F1 also reduced the levels of mRNA encoding HIF2α (Fig. 2D) but not that encoding HIF1α. E2F1 depletion also resulted in reduced HIF2α protein levels but no effect was seen in the protein levels of HIF1α or HIF1β (Fig. 2D). Similar results were also observed in additional cell lines, such as 786-O, U2OS and HepG2 (data not shown). This result suggests that E2F1 can regulate HIF2α transcription. To determine if this occurs through a direct mechanism – i.e. E2F1 is present at the promoter region of the HIF2α gene – chromatin immunoprecipitation (ChIP) assays, using an antibody specific for E2F1 were performed. Two sets of primers were designed that amplified the two regions with predicted consensus sequences for E2F1. This analysis revealed that E2F1 is present in the promoter region of HIF2α (Fig. 2E). Given that we found that E2F1 binds to both of these sites in the HIF2α promoter, we next determined the importance of each of these sites in controlling transcription of the HIF2α gene. To this end, we performed luciferase assays in which both sites were present, in which each of the sites were individually mutated, or in combination (Fig. 2F). This analysis revealed that when each of the sites were mutated, luciferase activity was significantly reduced, indicating that both sites are important. Furthermore, mutation of both sites at the same time did not further decrease luciferase activity when compared with the single mutants, further indicating that both sites are equally important for the regulation of the HIF2α promoter (Fig. 2F). Taken together, these analyses demonstrate that E2F1 is important for the direct regulation of expression of the HIF2α gene.

### Cezanne regulates HIF2α transcript through E2F1

**Fig. 3.**
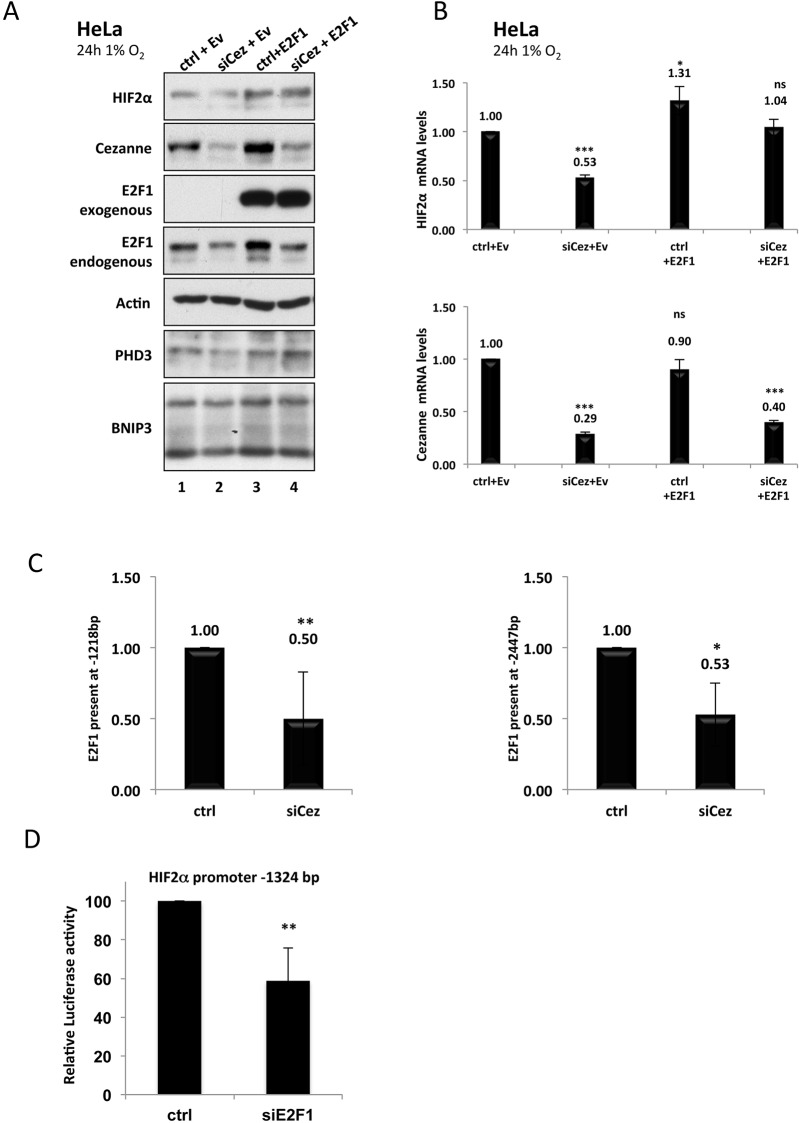
**Overexpression of E2F1 rescues HIF2α expression and activity in Cezanne-depleted cells.** (A) HeLa cells were co-transfected with 30 nM of either control (ctrl) or Cezanne-targeting siRNA (siCez) plus 1 µg of either empty vector (Ev) or E2F1 expression plasmid. At 24 h after transfection, cells were exposed to 1% O_2_ and incubated for a further 24 h. Whole cell lysates were analysed by western blotting with the antibodies indicated. (B) Cells were treated as explained in A, but total RNA was extracted, and the mRNA levels of HIF2α and Cezanne were determined by using RT-qPCR (*P*-values are significant according to the Student's *t*-test; ns, not significant, *P<0.05, ***P<0.001). The fold-changes relative to the control are shown above the bars. (C) HeLa cells were transfected with control or Cezanne-targeting siRNAs before fixation and lysis. ChIP analysis was performed using an antibody against E2F1 and control IgG. HIF2α promoter regions were amplified using specific primers, and the levels of E2F1 recruitment were analysed by using qPCR (*P-*values are significant according to the Student's *t*-test; *P<0.05, **P<0.01). (D) HeLa-HIF2α promoter luciferase cells were transfected with 30 nM of either control or E2F1-targeting siRNA for 48 h before lysis and luciferase activity analysis (*P*-values are significant according to the Student's *t*-test; **P<0.01).

Our results suggest that Cezanne-dependent stabilisation of E2F1 is required for HIF2α expression. To firmly verify this possibility, we overexpressed E2F1 in cells that had been depleted of Cezanne. The overexpression of E2F1 was sufficient to restore the levels of E2F1-dependent cyclin E expression (supplementary material Fig. S1C). Under these conditions, both the mRNA and protein levels of HIF2α could be rescued (Fig. 3A,B). Furthermore, the induction of the HIF targets PHD3 and BNIP3 was also restored (Fig. 3A). To further validate these results, we repeated our ChIP analysis for the HIF2α promoter in the presence or absence of Cezanne (Fig. 3C). This analysis revealed that in the absence of Cezanne there was a reduction in the presence of E2F1 at both of the binding sites that we had previously identified on the HIF2α promoter. In addition, we analysed the activity of a luciferase construct containing a portion of the HIF2α promoter, which included the −1218-bp site, in the presence or absence of E2F1 (Fig. 3D). This revealed that depletion of E2F1 reduced promoter activity significantly and to levels very similar to those obtained when we specifically analysed the E2F1 sites (Fig. 2F).

### Cezanne regulates E2F1 protein levels

**Fig. 4.**
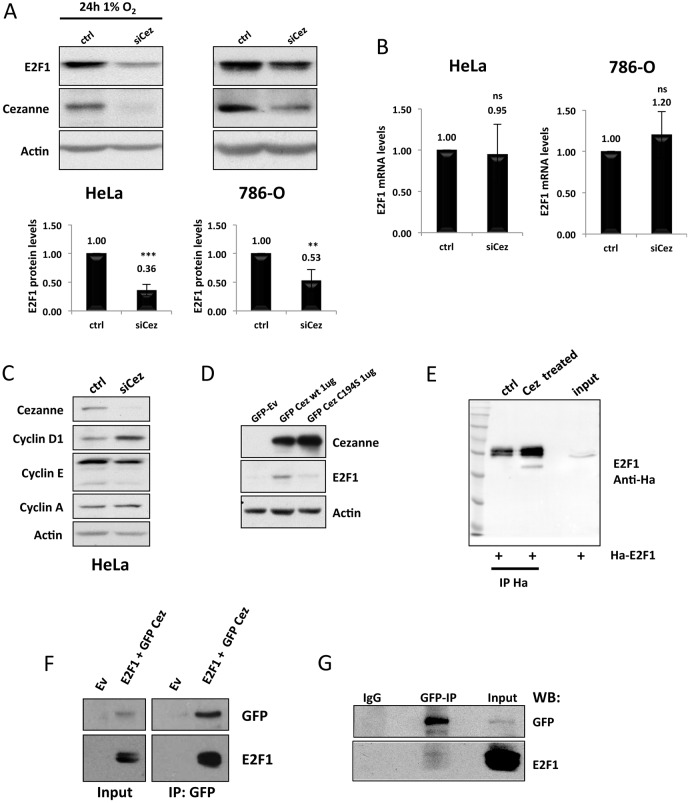
**Cezanne regulates E2F1 protein stability.** (A) HeLa and 786-O cells were transfected with either a non-targeting control (ctrl) or a Cezanne-targeting siRNA (siCez). At 24 h after transfection, HeLa cells were exposed to 1% O_2_, and whole cell lysates of both HeLa and 786-O cells were prepared after a further 24-h incubation. Whole cell extracts were analysed by western blotting to assess total levels of E2F1 and Cezanne. The band intensities were measured, and the values were normalised to those in cells transfected with the control siRNA (*P*-values are significant according to the Student's *t*-test; ***P<0.001). The fold-changes relative to the control are shown above the bars. (B) HeLa and 786-O cells were treated as in A, but the total RNA was extracted, and the transcript levels of E2F1 were analysed by using RT-qPCR. ns, not significant. (C) Whole cell extracts from HeLa cells that had been treated as described in A were analysed by western blotting with the antibodies indicated. (D) HeLa cells were transfected with either a wild-type (wt) or catalytically inactive (C194S) GFP–Cezanne construct, or with empty vector (Ev), and whole cell lysates were analysed by western blotting for the total E2F1 protein levels 48 h post transfection. (E) HEK293 cells were transfected with 5 µg of a HA–E2F1 construct for 48 h before lysis. Following immunoprecipitation (IP) with HA-beads, samples were treated where indicated with recombinant Cezanne (Cez). Lysates were analysed by western blotting with the indicated antibodies. (F) HEK293 cells were co-transfected with 5 µg of wild-type GFP–Cezanne and HA–E2F1 plasmids for 48 h before lysis. GFP–Cezanne was immunoprecipitated with an anti-GFP antibody, and lysates were analysed by western blotting with the indicated antibodies. (G) HEK293 cells were transfected with 5 µg of plasmid encoding wild-type GFP–Cezanne and left to express for 48 h. Cells were then lysed in a mild NP-40 lysis buffer, and GFP–Cezanne was immunoprecipitated with an anti-GFP antibody. Co-immunoprecipitation of endogenous E2F1 was analysed by western blotting (WB) as indicated.

The decrease in E2F1 protein levels in cells that had been depleted of Cezanne was also observable in 786-O cells (Fig. 4A). However, both in HeLa and in 786-O cells, there was no reduction in the mRNA levels of E2F1 (Fig. 4B), indicating that Cezanne interferes directly with E2F1 protein levels. This reduction of E2F1 protein had direct consequences on the expression of the E2F1-dependent target cyclin E, with that of cyclin D1 being slightly increased and that of cyclin A remaining unchanged (Fig. 4C). This indicates that Cezanne depletion has important implications for E2F1-dependent functions in the cell. In addition, we also determined that overexpression of Cezanne induced an increase in E2F1 protein levels, whereas expression of a catalytically inactive mutant (Bremm et al., 2014) did not (Fig. 4D).

E2F1 has been previously shown to be modified through K11-linked chains and to be targeted for proteasomal degradation through the E3 ligase anaphase-promoting complex/cyclosome (APC/C) with the activator protein Cdh1 (APC/C^Cdh1^) (Budhavarapu et al., 2012). The amount of ubiquitylated E2F1 is small, making it difficult to detect in our experiments (supplementary material Fig. S2C). As such, we could not detect the effects of Cezanne on the ubiquitylated form of E2F1; however, we still attempted ubiquitin chain restriction analysis (UbiCRest) (Hospenthal et al., 2015). This techniques allows the analysis of the effects of the deubiquitylase activity of Cezanne on E2F1 *in vitro*. Although, again, we could not detect the ubiquitylated form of E2F1, treatment of the samples with recombinant Cezanne resulted in increased levels of E2F1 (Fig. 4E). Therefore, our data point to E2F1 as a newly identified substrate for Cezanne. In support of this hypothesis, we observed that E2F1 co-immunoprecipitated with Cezanne, when E2F1 and Cezanne were overexpressed in HEK293 cells (Fig. 4F). Endogenous E2F1 could also be detected to interact with overexpressed Cezanne in cells (Fig. 4G). However, E2F1 levels following depletion of Cezanne could not be rescued by proteasomal inhibition (supplementary material Fig. S2D–F), indicating that the degradation pathway that is elicited through Cezanne depletion is distinct from that mediated by APC/C^Cdh1^.

### Cezanne depletion results in defective cell cycle progression

**Fig. 5.**
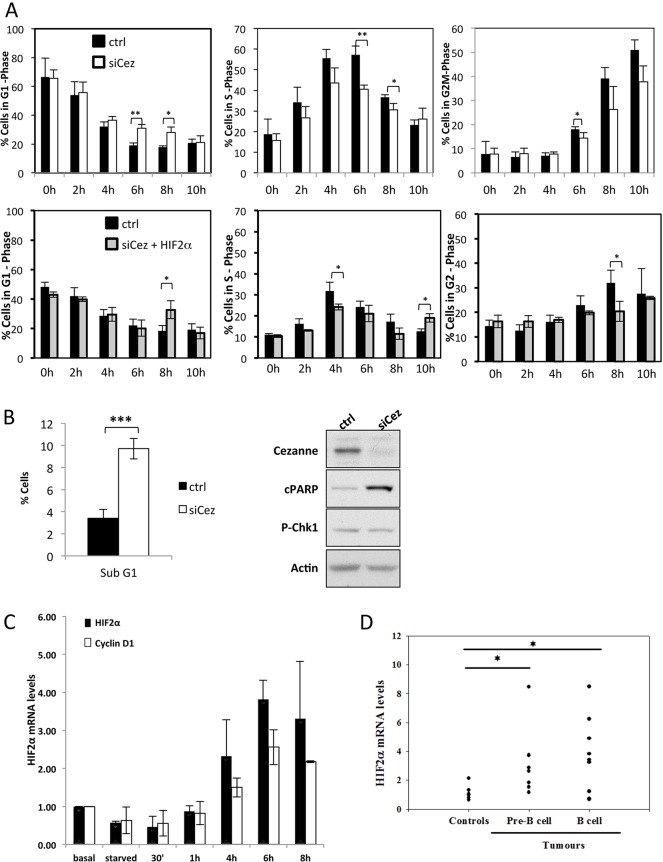
**Cezanne depletion causes defects in cell cycle progression, and HIF2α mRNA responds to mitogenic and oncogenic signals.** (A) HeLa cells were transfected with control (ctrl), Cezanne-targeting siRNAs (siCez), or siCez and HIF2α-targeting siRNA (siCez+HIF2α) before harvesting for cell cycle analysis, using the double thymidine block protocol (*P*-values are significant according to the Student's *t*-test; *P<0.05, **P<0.01). (B) Left panel, HeLa cells were treated and analysed as described in A, and the total percentage of cells across the multiple time points shown in A for control or Cezanne-depleted conditions was calculated (*P*-values are significant according to the Student's *t*-test; ***P<0.001); right panel, whole cell lysates from HeLa cells transfected with either control or Cezanne-targeting siRNAs were analysed by western blotting with the antibodies indicated. (C) HeLa cells were grown in reduced-serum medium for 24 h, and after replenishing with medium containing 10% FBS, harvested for mRNA analysis at various time points. Cyclin D1 was used as a positive control for growth-factor-dependent gene expression induction. (D) Wild-type B lymphocytes and Eµ-Myc-derived pre-B and B-cell tumours were analysed for HIF2α mRNA levels by using RT-qPCR. The graph depicts the levels obtained for each mouse, which were analysed and compared to a control wild-type level (*P*-values are significant according to the Student's *t*-test; *P<0.05).

Although E2F1 is required for cell cycle progression and the presence of K11-linked chains has been associated with progression through S-phase and mitosis (Matsumoto et al., 2010), the role of Cezanne in regulating the cell cycle is still unclear. We thus performed cell synchronisation–release experiments in HeLa cells using double thymidine block to arrest the cells in the G1 phase and flow cytometry to analyse the cell cycle profile. Although, the synchronisation was successful in both control and Cezanne-depleted cells, progression into S-phase was defective in the absence of Cezanne. When compared with siRNA control cells, 4–6 h post-release, Cezanne-depleted cells were defective in their progression into S-phase and a higher proportion of these cells remained in the G1 phase (Fig. 5A). A similar impairment was also observed in the proportion of cells in G2/M phase. These results are consistent with a defect in entry into S-phase when levels of Cezanne are reduced. Depletion of HIF2α did not change cell cycle progression significantly (supplementary material Fig. S3A), and co-deletion of Cezanne and HIF2α only produced a small change in the timing of delay observed in cell cycle progression (Fig. 5A).

Interestingly, and in agreement with our previous finding under hypoxia (Bremm et al., 2014), Cezanne-depleted cells have higher levels of cell death and increased markers of apoptosis under normoxia (Fig. 5B), possibly indicative of defects in S-phase entry.

Our analysis revealed that HIF2α is an E2F1 target, suggesting that HIF2α expression can be regulated through the cell cycle and in response to mitogenic signals. As such, we tested whether HIF2α mRNA could be induced by growth factors, known stimuli for cell cycle progression. HeLa cells were starved for 24 h in medium containing 0.5% fetal bovine serum (FBS) and then harvested for mRNA analysis at several time points after replenishing the cells with full serum. Interestingly, HIF2α mRNA levels were strongly induced after 4 h of medium replacement, indicating a role for growth factors in HIF2α regulation (Fig. 5C). Cyclin D1 was used as a control for growth factor stimulation, and its activation profile was very similar to that of HIF2α. These data support the hypothesis that Cezanne can regulate HIF2α expression in an E2F1-dependent manner and suggest that HIF2α is responsive to cell cycle inducers, such as mitogenic signals.

HIF2α has been shown to cooperate with the cell cycle regulator and transcription factor Myc (Gordan et al., 2007b). By contrast, E2F1 has been shown to be required for Myc-mediated proliferation in a model of lymphoma in mice (Baudino et al., 2003). To determine whether HIF2α mRNA levels change in a model of Myc-induced lymphoma, such as the Eµ-Myc transgenic mouse, we analysed HIF2α mRNA levels in wild-type and Myc-overexpressing pre-B and B-cells (Fig. 5D). Our analysis revealed that Myc overexpression induced very high levels of the E2F1 target cyclin E, further indicating activation of the E2F family of transcription factors in this model (supplementary material Fig. S3B). Interestingly, oncogenic Myc signalling in this model resulted in an induction of HIF2α mRNA in both pre-B and B cells that had been isolated from Eµ-Myc mice compared with those from wild-type mice (Fig. 5D). These results further support our model that engagement of the E2F pathway leads to expression of the HIF2α gene.

## DISCUSSION

The occurrence of hypoxia in many tumours is associated with increased resistance to chemo- and radiotherapy, as well as with a poor prognosis (Semenza, 2012). In addition, the deregulated expression of HIF transcription factors has been reported in several tumour types and thus far no effective therapies are known to target HIF-dependent effects (Keith et al., 2012). Given the range of signalling and metabolic pathways that are regulated by HIF and that contribute to tumour progression, it is highly relevant to have a more detailed understanding of how HIF expression and turnover are regulated. Furthermore, considering the specificity and often tumour-promoting effects of HIF2α, it is necessary to have a better understanding of how this subunit is regulated when designing new drugs to target HIF activity.

Here we describe the regulation of HIF2α protein levels and activity by a deubiquitylase, Cezanne, and show that Cezanne regulates HIF2α gene expression by modulating the protein levels of E2F1, an important transcription factor associated with cell cycle progression (Stevens and La Thangue, 2003).

So far, there is only one report on the regulation of the HIF2α promoter. Wada and collaborators report that HIF2α expression is modulated by SP1 and SP3 during adipogenesis (Wada et al., 2006) in an adipocyte cell model. We show here that E2F1 is a transcription factor that is required for the expression of HIF2α in a diverse range of cell lines, such as HeLa and 786-O cells, indicating that E2F1 is a general regulator of HIF2α expression. This hypothesis is supported by the observation that known activators of E2F1 activity, such as growth factors, are also capable of inducing expression of HIF2α. Our analysis revealed that HIF2α mRNA is responsive to oncogenic cellular (c)-Myc activation in a model of lymphoma. Interestingly, when analysing publicly available datasets of human cancers, HIF2α mRNA levels are substantially increased in lymphoma (Oncomine). In these analyses, four out of eight datasets demonstrated high levels of HIF2α mRNA when compared with the levels in normal, non-tumour cells (Oncomine). Given that c-Myc and E2F1 are potent cell cycle regulators, our observations additionally imply an as yet unexplored cell-cycle-dependent regulation of HIF2α gene expression.

The observation that Cezanne co-immunoprecipitates with E2F1 and that Cezanne depletion decreases the levels of E2F1, whereas a wild-type but not a catalytically inactive overexpressed Cezanne construct increased E2F1 protein, suggests that E2F1 is a newly identified substrate for Cezanne. This is further supported by the report that E2F1 is modified through K11-linked ubiquitin chains (Budhavarapu et al., 2012); *in vivo*, Cezanne is known to have preference for K11-linked chains (Bremm et al., 2014). We were unable to readily detect ubiquitylated E2F1 in untreated cells, indicating that only a small amount of E2F1 is ubiquitylated at specific stages of the cell cycle or that this is rapidly degraded. Because proteasome inhibition did not rescue E2F1 levels following Cezanne depletion, we could not stabilise the ubiquitylated form to significant levels either. However, using the UbiCRest analysis of E2F1, where recombinant Cezanne was added to E2F1 that had been recovered from cells, we could again detect increased levels of E2F1, further indicating that E2F1 is a newly identified substrate for Cezanne.

The role of Cezanne in cell cycle regulation has not been truly explored as of yet, and an initial report indicates that Cezanne depletion is not associated with proliferation defects (Neumann et al., 2010). However, we observed that Cezanne-depleted cells cannot progress normally through the cell cycle, which is most likely owing to the downregulation of E2F1 protein levels and not to HIF2α. However, given the preference of Cezanne for K11-linked chains (Bremm et al., 2010, 2014) and the cell cycle distribution of these chains (Matsumoto et al., 2010; Meyer and Rape, 2014), it is very likely that additional substrates exist in different stages of the cell cycle. Other approaches using synchronisation techniques at different stages of the cell cycle – such as mitosis – could help to elucidate the effects of Cezanne during the cell cycle.

Although the regulation of HIF2α expression by Cezanne is most likely to be primarily at the transcription level, we cannot disregard the possibility of a direct effect on the HIF2α protein. We detected by using mass spectrometry that Cezanne and HIF2α can exist in a complex (supplementary material Fig. S4), and so it is possible that Cezanne regulates HIF2α directly, in a manner similar to that described for HIF1α (Bremm et al., 2014). However, given the ability of E2F1 to rescue HIF2α levels in the absence of Cezanne, this implies that the direct regulation of HIF2α by Cezanne is minimal, at least in the cells systems we have analysed.

Cezanne has been associated with tumour progression, namely in breast cancer (Pareja et al., 2012), and it has also been linked to deregulated NF-κB signalling in the context of inflammation (Enesa et al., 2008; Hu et al., 2013; Luong et al., 2013). The effect we now report on E2F1 protein levels indicates a broader impact of this deubiquitylase in cancer through its impact on cell cycle regulation. Taken together with the regulation of HIF1α expression that we have reported previously, Cezanne appears to be an attractive new drug target in the treatment of cancer.

## MATERIALS AND METHODS

### Cell lines, treatments and transfections

Human cell lines HeLa and HEK293 were cultured in Dulbecco's modified Eagle's medium (DMEM), and cell line 786-O was cultured in RPMI Medium 1640 (Lonza) supplemented with 10% (v/v) FBS (Gibco), l-Glutamine (Gibco), 50 U/ml penicillin and 50 μg/ml streptomycin (Gibco) at 37°C under 5% CO_2_. Extracts from murine cells were derived from cohorts housed at the Cancer Research UK Beatson Institute and covered by the University of Glasgow ethical review process and project licence PPL60/4181. Wild-type mice were used as control. All mice were on a C57Bl/6J background. CD19-positive cells were isolated from the lymph nodes and pelleted before RNA extraction. HeLa-HIF2α promoter cells were generated by transfecting HeLa cells with a commercially purchased HIF2α *Renilla* luciferase promoter and a puromycin resistance cassette in the ratio of 1:9. Transfected cells were selected using 2 μg/ml puromycin (Sigma) 48 h after transfection. Once selection was complete, cells were maintained in complete DMEM supplemented with 0.5 μg/ml puromycin.

#### Hypoxia

Hypoxia at 1% O_2_ was achieved using an INVIVO2 hypoxia workstation (Ruskinn, Bridgend, Wales). To avoid reoxygenation cells were lysed inside the workstation.

#### Proteasome inhibition

Cells were treated with 10 µM or 20 µM MG132 (Merck-Millipore) for 3 or 7 h as indicated. Two additional proteasomal inhibitors were used in HeLa cells, and the treatments were with 10 µM MLN9708 (Stratech Scientific) for 1 h or 2 µM Epoxomicin (Merck-Millipore) for 4 h.

#### Proline hydroxylase inhibition

Cells were treated with DMOG (1 mM final concentration), or DFX mesylate (Sigma) was added at a final concentration of 200 µM for 1 h 30 min and 24 h, respectively.

#### Growth factors

To test the effects of growth factors on HIF2α expression, HeLa cells were incubated for 24 h in medium containing 0.5% of FBS and then harvested at the different time points after medium replacement containing 10% FBS.

#### Plasmids

GFP-Cezanne wild type and the C145S mutant have been described previously (Bremm et al., 2014), E2F1-ER plasmid was a kind gift from Dr Victoria Cowling (University of Dundee, Dundee, UK). The HRE-luciferase construct was a kind gift from Professor Giovanni Melillo (Astra Zeneca, Gaithersburg, MA). Ha-E2F1 (Addgene 24225) was a gift from Professor Kristian Helin (Lukas et al., 1996). The HIF2α promoter construct was obtained from Switchgear genomics. HIF2α E2F1 sites were cloned using *Kpn*I and *Mlu*I restriction enzymes in the pGL3-vector luciferase construct (Promega) using the following oligonucleotides – wild-type E2F1 forward 5′-CTGCCCTTTTCCCGCACTCTAGCATCCCCGCCAAAACCAAACA-3′, reverse 5′-CGCGTGTTTGGTTTTGGCGGGGATGCTAGAGTGCGGGAAAAGGGCAGGTAC-3′; E2F1mut1 (−2447 bp) forward 5′-CTGCCCTCAAAAAGCACTCTAGCATCCCCGCCAAAACCAAACA-3′, reverse 5′-CGCGTGTTTGGTTTTGGCGGGGATGCTAGAGTGCTTTTTGAGGGCAGGTAC-3′; E2F1mut2 (−1218 bp) forward 5′-CTGCCCTTTTCCCGCACTCTAGCATCCCTAAAACCCACCAAACA-3′; reverse 5′-CGCGTGTTTGGTGGGTTTTAGGGATGCTAGAGTGCGGGAAAAGGGCAGGTAC-3′; E2F1mut1 and E2F1mut2 forward 5′-CTGCCCTCAAAAAGCACTCTAGCATCCCTAAAACCCACCAAACA-3′, reverse 5′-CGCGTGTTTGGTGGGTTTTAGGGATGCTAGAGTGCTTTTTGAGGGCAGGTAC-3′.

#### siRNA transfections

siRNA duplex oligonucleotides were synthesized by MWG Eurofins – control siRNA (5′-CAGUCGCGUUUGCGACUGG-3′); against Cezanne (5′-CCGAGUGGCUGAUUCCUAU-3′), against HIF2α (5′-CAGCAUCUUUGACAGU-3′) and against E2F1 (5′-CGCUAUGAGACCUCACUG-3′).

Cells (2×10^5^) were seeded in 6-well plates and transfected after 24 h with 30 nM siRNA duplexes using INTERFERin transfection reagent (Polyplus). siRNA and DNA co-transfections were performed using 30 nM siRNA plus 1 µg of plasmid, using jetPRIME (Polyplus), according to the manufacturer's instructions.

#### Immunoprecipitation

For immunoprecipitation experiments, 5 μg of plasmid DNA encoding wild-type GFP–Cezanne per 10-cm dish was transiently transfected into HEK293 cells by using calcium phosphate, as described previously (Webster and Perkins, 1999). For gain-of-function experiments, 1 µg of each plasmid DNA GFP–Cezanne (wild type or C194S) or E2F1 per 3.5-cm dish was transiently transfected, using jetPRIME (Polyplus), according to the manufacturer's instructions.

#### Cell cycle analysis

Cell cycle analysis was performed following a cell synchronisation–release protocol using treatments with thymidine to block cell cycle progression in the G1-S phase of the cell cycle. Cells were transfected as described above with either a control or a Cezanne-specific siRNA oligonucleotide. At 24 h post-transfection, cells were washed three times with PBS and incubated for 19 h with medium that had been supplemented with a 2 mM final concentration of thymidine. Next, cells were PBS-washed again and released for 9 h into normal growth medium. After a second incubation with thymidine for 15 h, cells were once more released and harvested every 2 h, for a total of 10 h, by using trypsin detachment and ethanol fixation, and cells were then stored at −20°C. Cell cycle profile analysis was performed in a Guava^®^ easycyte HT (Millipore) apparatus, using the Guava^®^ Cell Cycle Reagent (Millipore 4500-0220), according to the manufacturer's instructions.

### Luciferase reporter assay

Cells (2×10^5^) were seeded in six-well plates and transfected with 30 nM siRNA duplexes using INTERFERin transfection reagent (Polyplus). At 48 h post-transfection, cells were lysed in 400 µl passive lysis buffer (Promega). For E2F1 sites, cells were transfected with 1 µg of luciferase constructs for 48 h before lysis. Luciferase assays were performed according to the manufacturer's instructions (Luciferase Assay System, Promega). Results were normalised for protein concentration with all experiments being performed a minimum of three times before calculating means and standard deviations, as shown in figures.

### Immunoblot

Cells were lysed in RIPA buffer [50 mM Tris-HCl (pH 8), 150 mM NaCl, 1% (v/v) NP40, 0.5% (v/v) Na-deoxycholate, 0.1% (v/v) SDS] with 1 tablet/10 ml Complete Mini EDTA-free protease inhibitors (Roche). SDS-PAGE and immunoblots were performed using standard protocols.

Antibodies against the indicated proteins were used as follows – HIF2α (PA1-16510, Thermo Scientific), Cezanne (custom antibody, Eurogentec), β-actin (3700, Cell Signaling), PHD3 (A300-327A, Bethyl Labs), BNIP3 (ab10433, Abcam), Glut-1 (53519, Anaspec), p52 (05-361, Merck Millipore), E2F1 (3742, Cell Signaling), SP1 (07-645, Upstate-Millipore), cyclin D1 (DCS6, Cell Signaling), cyclin E (HE12, Cell Signaling), cyclin A (C-19, Santa Cruz), GFP (2956, Cell Signaling), cleaved PARP (D214) (9541, Cell Signaling), c-Myc (9E10, Sigma), phosphorylated Chk1 at S345 (2341, Cell Signaling).

### Immunoprecipitation

For immunoprecipitation of endogenous E2F1, HEK293 cells were transiently transfected with GFP–Cezanne and subsequently lysed in 200 μl lysis buffer per 10-cm dish [10 mM Tris-HCl (pH 7.5), 150 mM NaCl, 1% (v/v) Triton X-100, 20% (v/v) glycerol] with 1 tablet/10 ml of Complete Mini EDTA-free protease inhibitors (Roche).

Cleared cell lysate was rotated overnight at 4°C with 2 μg of anti-E2F1 antibody and then for an additional 1 h 30 min after adding protein-G–Sepharose (Generon). Immobilized antigen–antibody complex was then washed three times with PBS and eluted in 20 µl Laemmli buffer (2× SDS buffer) buffer.

For mass spectrometry analysis, three 10-cm dishes of HeLa cells, with an optical confluence of 80–90%, were incubated for 24 h at 1% O_2_ before harvesting with a detergent-free lysis buffer (50 mM Tris-HCl pH 8, 150 mM NaCl, 2 mM EDTA, 1 mM DTT). After 15 min incubation on ice, the lysates were passed through a 25 G syringe five times and cleared by centrifugation. Immunoprecipitation was performed using an anti-HIF2α antibody [EPAS-1 (A-5), Santa Cruz].

The mass-spectrometry-based immunoprecipitation experiments were performed in triplicate. Protein preparations were separated by using an SDS-PAGE gel, fractionated into eight fractions and an in-gel digestion was performed. The samples were reduced and alkylated with DTT and iodoacetamide, and digested using sequencing-grade trypsin (Roche). The resulting peptides were then cleaned over a C18 column, and submitted for mass spectrometric analysis.

The samples were run on the Orbitrap Velos (Thermo Fisher) using a 180-min gradient (10–40% acetonitrile, 80% acetonitrile and 2% Formic acid). The parent ion scan was 350–1800 Da, at 60,000 resolution. The tandem mass spectrometry (MS/MS) scan was performed with a minimum signal of 5000, default charge state of >2, on the top 10 ions, with a normalised collision energy of 35. Dynamic exclusion of 120 s was utilised.

The resulting mass spectrometry raw data was processed using MaxQuant version 1.3.0.3 (Cox and Mann, 2008), using the Andromeda search engine against the Uniprot Human database (2012). The variable modifications were oxidation (M), deamidation (NQ) and acetylation (protein N-terminus), with a fixed modification of carbamidomethyl (C). The peptide and protein FDR was set to 0.01. Protein identifications with <2 peptides, identified as contaminants (as designated by MaxQuant), a PEP value of >0.05 and reversed sequence hits were excluded from further analysis.

### Immunoprecipitation and UbiCRest of HA-tagged E2F1

HEK293 cells (3×10^6^) were seeded in 10-cm culture dishes and transfected 24 h later with 5 µg pCMV-HA-E2F1. Subsequently, cells were lysed in 50 mM Tris-HCl (pH 7.4), 150 mM NaCl, 1% (v/v) IGEPAL^®^ CA-630, 0.2% (v/v) SDS, 10 mM NaF, 1 mM PMSF, 5 mM N-ethylmaleimide, 1× Complete EDTA-free protease inhibitors (Roche) and 1 µl/ml Benzonase^®^ Nuclease (≥250 units/µl). Cleared lysates were incubated with 10 µl of agarose conjugated to an antibody against hemagglutinin (HA) (Sigma-Aldrich #A2095) per sample for 3 h at 4°C. Beads were washed three times with wash buffer [50 mM Tris-HCl (pH 7.4), 150 mM NaCl], once with DUB buffer [50 mM Tris-HCl (pH 7.4), 50 mM NaCl, 5 mM DTT] and equally split in two reaction tubes. Activation of 2 µg GST-Cezanne^1-449^ was initiated by incubating in DUB dilution buffer [150 mM NaCl, 25 mM Tris (pH 7.4), 10 mM DTT] for 10 min at room temperature. GST–Cezanne or DUB buffer alone (control) was added to immobilised HA–E2F1 and incubated for 1 h at 37°C. Subsequent SDS-PAGE and immunoblots were performed using standard protocols.

### Analysis of gene expression levels by using reverse transcriptase RT-PCR

RNA was extracted using peqGOLD total RNA kit (Peqlab), according to the manufacturer's instructions, and reverse transcribed using QuantiTect Reverse Transcription Kit (Qiagen). For quantitative (q)PCR, Brilliant II Sybr green kit (Stratagene-Agilent), including specific MX3005P 96-well semi-skirted plates, were used to analyse samples on the MX3005P qPCR platform (Stratagene-Agilent). Actin was used as a normalising agent in all experiments. The following primers were used for RT-PCR (the prefix ‘m’ denotes ‘mouse’) – actin F 5′-CTGGGAGTGGGTGGAGGC-3′, R 5′-TCAACTGGTCTCAAGTCAGTG-3′; HIF2α F 5′-TTTGATGTGGAAACGGATGA-3′, R 5′-GGAACCTGCTCTTGCTGTTC-3′; Cezanne F 5′-ACAATGTCCGATTGGCCAGT-3′, R 5′-ACAGTGGGATCCACTTCACATTC-3′; cyclin D1 F 5′-AGTCCGTGTGACGTTACTGTTGT-3′, R 5′-CTCCCGCTCCCATTCTCT-3′; E2F1 F 5′-ATGTTTTCCTGTGCCCTGAG-3′, R 5′-ATCTGTGGTGAGGGATGAGG-3′; mActin F 5′-ATGCTCCCCGGGCTGATAT-3′, R 5′-CATAGGAGTCCTTCTGACCCATTC-3′; mHIF2α F 5′-ATCACGGGATTTCTCCTTCC-3′, R 5′-GGTTAAGGAACCCAGGTGCT-3′; mCyclin E F 5′-CTGGACTCTTCACACAGATGAC-3′, R 5′-GCCTATCAACAGCAACCTACA-3′.

### Chromatin immunoprecipitation

ChIP was performed using an adaptation of Schumm and colleagues' method (Schumm et al., 2006). Proteins and chromatin were cross-linked with 1% formaldehyde at room temperature for 10 min. Glycine was added to a final concentration of 0.125 M for 5 min to quench the reaction. Cells were harvested into 400 µl of lysis buffer (1% SDS, 10 mM EDTA, 50 mM Tris-HCl pH 8.1, 1 mM PMSF, 1 μg/ml leupeptin, 1 μg/ml aprotinin) and left on ice for 10 min. Samples were then sonicated at 4°C eight times for 15 s with a 30-s gap between each sonication at 50% amplitude (Sonics Vibra-Cell, number VCX130). Supernatants were recovered by using centrifugation (13,000 ***g*** for 10 min at 4°C) before 10% of each sample was stored as input. Remaining samples were split into 120-µl aliquots before being diluted tenfold in dilution buffer (1% Triton X-100, 2 mM EDTA, 150 mM NaCl, 20 mM Tris-HCl pH 8.1). Diluted samples were pre-cleared for 2 h at 4°C by incubating with 2 µg of sheared salmon sperm DNA and 20 µl of protein G-Sepharose (50% slurry).

Immunoprecipitations were performed overnight on the remaining sample with 2 µg of anti-E2F1 antibody, with the addition of Brij 35 detergent to a final concentration of 0.1%. Immune complexes were captured by incubating with 40 µl of protein-G–Sepharose (50% slurry) and 2 µg salmon sperm DNA for 1 h at 4°C. The immunoprecipitates were washed sequentially for 5 min each at 4°C in Wash Buffer 1 (0.1% SDS, 1% Triton X-100, 2 mM EDTA, 20 mM Tris-HCl pH 8.1, 150 mM NaCl), Wash Buffer 2 (0.1% SDS, 1% Triton X-100, 2 mM EDTA, 20 mM Tris-HCl, pH 8.1, 500 mM NaCl) and Wash Buffer 3 (0.25 M LiCl, 1% Nonidet P-40, 1% deoxycholate, 1 mM EDTA, 10 mM Tris-HCl pH 8.1). Beads were washed twice with Tris-EDTA buffer and eluted with 120 µl of Elution Buffer (1% SDS, 0.1 M NaHCO_3_). Cross-links were reversed by incubating with 0.2 M NaCl at 65°C overnight and Proteinase K (20 µg each), 40 mM Tris-HCl pH 6.5 and 10 mM EDTA for 1 h at 45°C was used to remove protein. DNA was purified using a PCR-product purification kit according to the manufacturer's instructions (NBS Biologicals, number NBS363). A 3-µl aliquot of DNA was used for qPCR with the following primers for the HIF2α promoter (−2447 bp or −1218 bp) – HIF2α promoter (−1218 bp) F 5′-CCCTCGCTTTCCAACTTCAA-3′, R 5′-CGCCTACTCTTCCTTCCCTC-3′; HIF2α promoter (−2447 bp) F 5′-TCTTGAGTGACCCCTCCTTG-3′, R 5′-CTCAAGTGATCTGCCCAACT-3′.

## Supplementary Material

Supplementary Material
