# Supplementary material for: Cezanne regulates E2F1-dependent HIF2α expression
Source: J Cell Sci. 2015 Aug 15;128(16):3082–93. doi: 10.1242/jcs.168864 (PMC4541044; doi:10.1242/jcs.168864)
Supplement: Supplementary Material [file supp_128_16_3082__index.html]

Supplementary Material 

# Cezanne regulates E2F1-dependent HIF2α expression

## JCS168864 Supplementary Material

- Supplementary Material
