# Supplementary material for: Cezanne regulates E2F1-dependent HIF2α expression
Source: J Cell Sci. 2015 Aug 15;128(16):3082–93. doi: 10.1242/jcs.168864 (PMC4541044; doi:10.1242/jcs.168864)
Supplement: Supplementary Material [file supp_jcs.168864_JCS168864supp.pdf]

Supplementary Figures

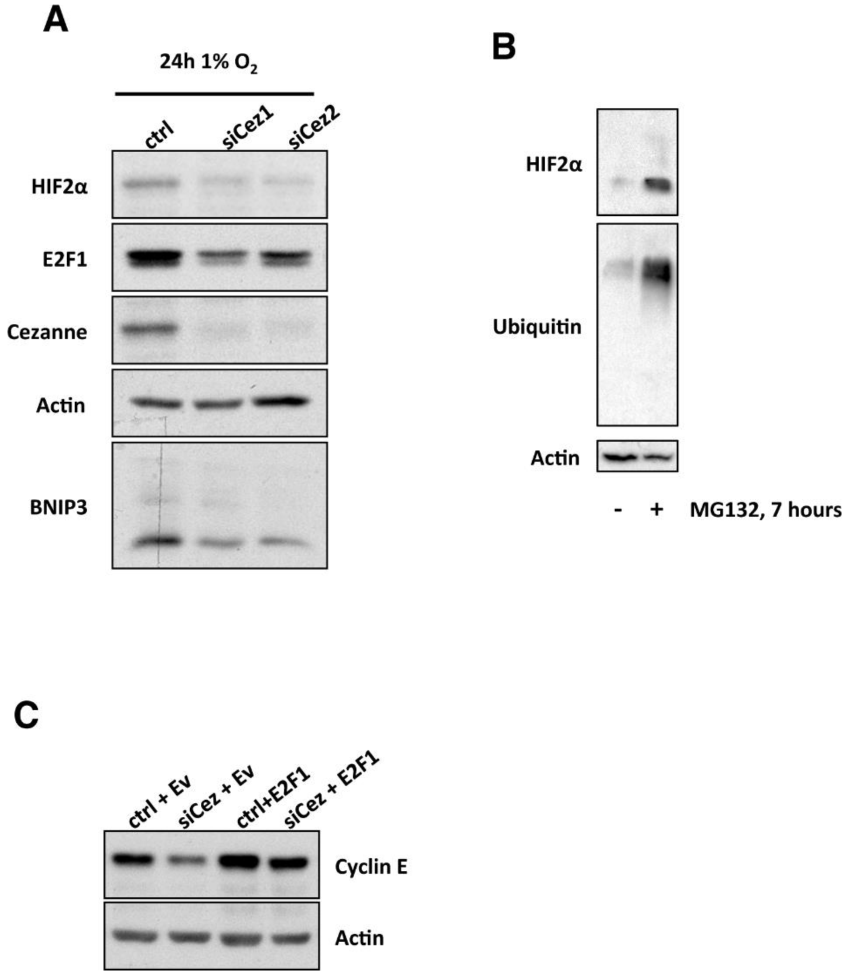

Supplementary Figure S1. Validation of the effect of Cezanne on HIF2α protein levels and overexpression of exogenous E2F1 can rescue E2F1 activity. A) HeLa cells were transfected with either control (ctrl) or Cezanne siRNAs and incubated for 24 h at 1% O_2. Whole cell lysates were analysed by western blot with the antibodies indicated. B) HeLa cells were treated where indicated with 10 μM MG132 for 7 hours prior to lysis in SDS. Whole cell lysates were analysed by western blot with the indicated antibodies. C) HeLa cells were co-transfected with 30nM of either control (ctrl) or Cezanne siRNA plus 1 μg of either empty vector or E2F1 plasmid. 24 h after transfection, cells were exposed to 1% O_2 and incubated for further 24h. Whole cell lysates were analysed by western blot with an anti-Cyclin E antibody as readout of E2F1 transcriptional activity.

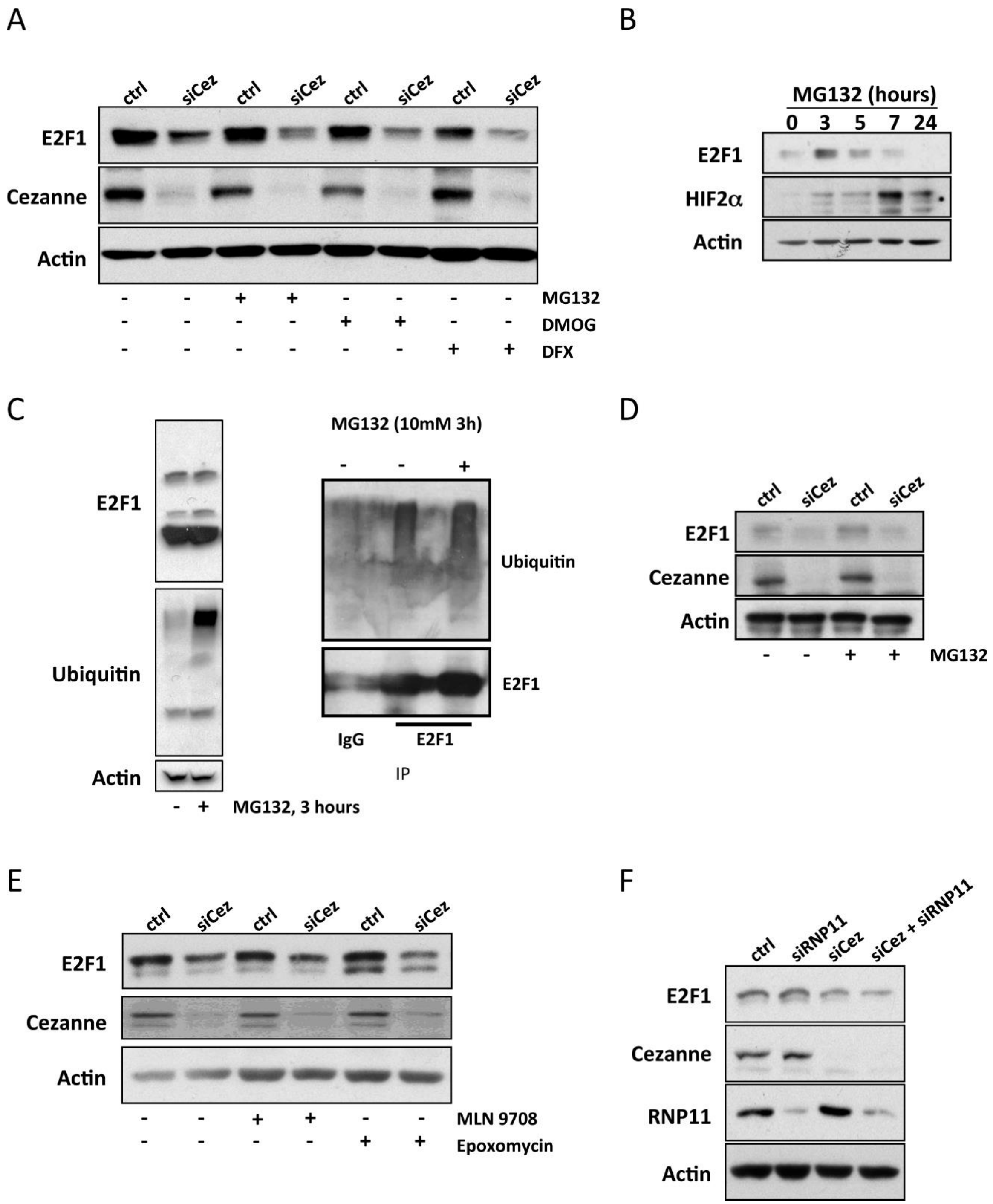
A
ctrl
siCez
ctrl
siCez
ctrl
siCez
ctrl
siCez
E2F1
Cezanne
Actin
MG132
DMOG
DFX
B
MG132 (hours)
0 3 5 7 24
E2F1
HIF2α
Actin
C
E2F1
Ubiquitin
Actin
- + MG132, 3 hours
MG132 (10mM 3h)
Ubiquitin
E2F1
IgG
E2F1
IP
D
ctrl
siCez
ctrl
siCez
E2F1
Cezanne
Actin
MG132
E
ctrl
siCez
ctrl
siCez
ctrl
siCez
E2F1
Cezanne
Actin
MLN 9708
Epoxomycin
F
ctrl
siRNP11
siCez
siCez + siRNP11
E2F1
Cezanne
RNP11
Actin

Supplementary Figure S2. Cezanne regulates E2F1 protein stability by a proteasomal degradation-independent mechanism. A) HIF proteasomal degradation pathways were inhibited in HeLa cells transfected with control (ctrl) or Cezanne siRNAs and exposed to 1% O_2 for 24 h, treated with 20 μM MG132 for 3 h, 1 mM DMOG for 90 min or 200 μM DFX for 24 h and E2F1 protein levels were analysed by western blot. B) HeLa cells were treated with 10 μM MG132 for the indicated periods of time prior to lysis. Western blots were performed analysing the levels of the indicated proteins C) HeLa cells were treated with 10 μM MG132 for 3 h prior to lysis in SDS. Western blots were performed analysing the levels of the indicated proteins. (left panel). 293 cells were transfected with empty vector or Ha-E2F1 prior to treatment with 10 μM MG132 for 3 h and lysis in SDS. After dilution, E2F1 was immunoprecipitated and analysed by western blot using the indicated antibodies (right panel). D) HeLa cells transfected with control (ctrl) or Cezanne siRNAs and treated with 10 μM MG132 prior to lysis and analysis by western blot for the levels of the indicated proteins. E) Cells extracts were prepared as in **(A)** except that cells were treated with 10 μM MLN9708 for 1 h or 2 μM Epoxomicin for 4 h. F) HeLa cells transfected with control (ctrl), RPN11, Cezanne or RPN11 and Cezanne siRNAs prior to lysis and analysis by western blot for the levels of the indicated proteins.

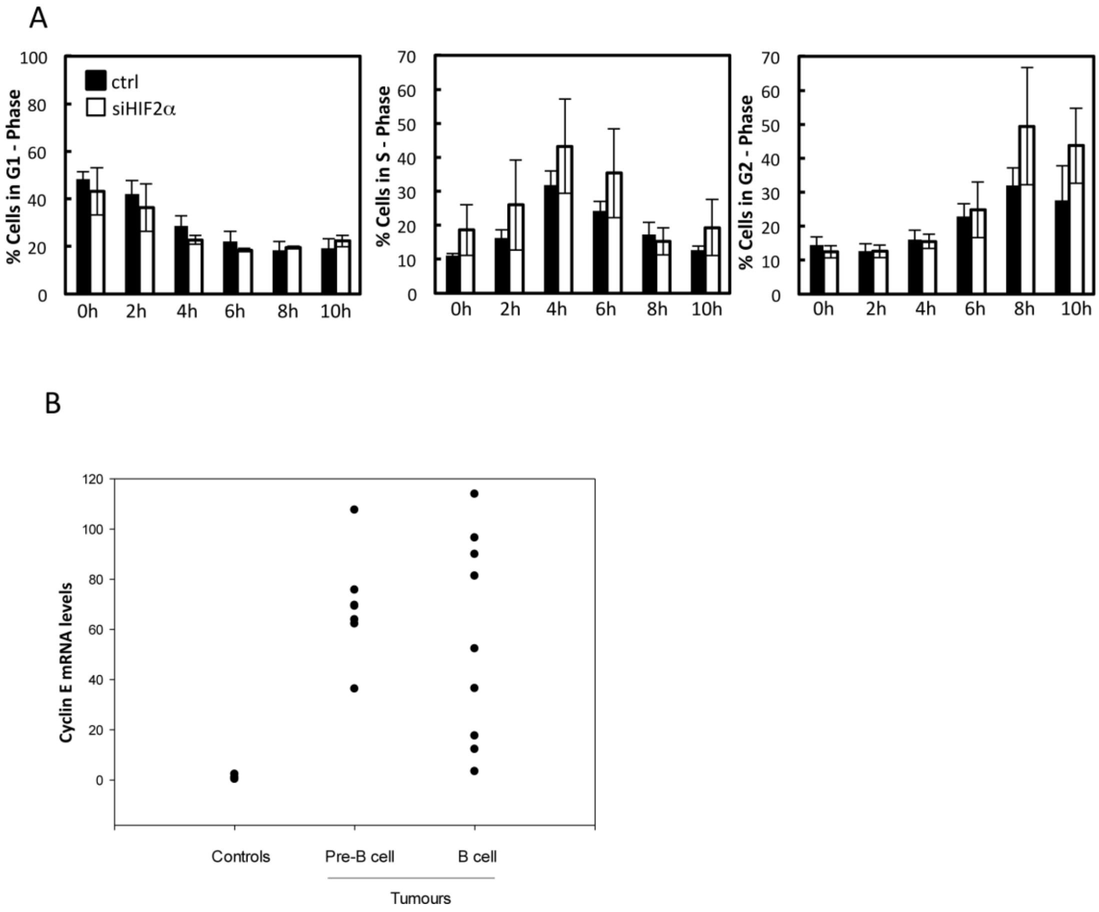

Supplementary Figure S3. HIF2α depletion does not alter cell cycle progression and Eu-Myc derived pre-B and B-cells have high levels of Cyclin E. A) HeLa cells were transfected with control (ctrl) or HIF2α siRNAs, prior to harvesting for cell cycle analysis, using the double thymidine block protocol. B) Wild type B lymphocytes and Eμ-Myc derived pre-B and B-cell tumours were analysed for Cyclin E mRNA levels by RT-qPCR. Graph depicts levels obtained for each mouse analysed and compared to wild type levels.

A

Protein Name	OTU domain-containing protein 7B; Zinc finger protein Cezanne
Gene Name	OTUD7B;ZA20D1;RP11-212K13.2-003;mCG_122520; Otud7b
Proteins	9
Peptides	3
PEP	5.42E-19
Sequence coverage	4.9%
Intensity	3352900
Ratio Intensities	3.24

B

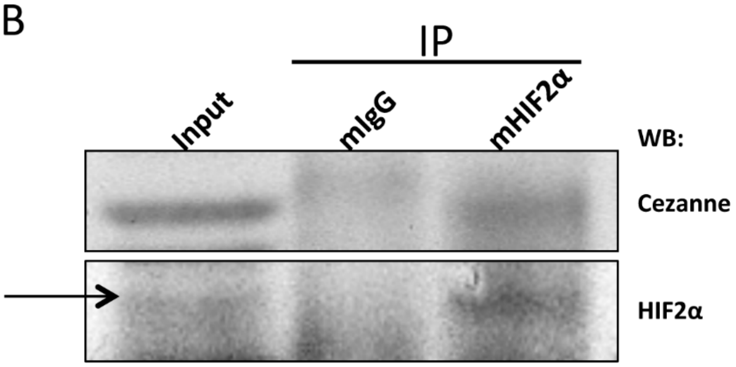

Supplementary Figure S4. The deubiquitinase Cezanne is identified as a potential binding partner for HIF2α. A) HIF2α was immunoprecipitated from HeLa cells incubated for 24 h in 1% O_2. Mass spectrometry data is presented. B) Data from the mass spectrometry analysis was validated by western blot analysis of co-immunoprecipitation of HIF2α with Cezanne.
